# A cost‐effectiveness analysis of avelumab plus best supportive care versus best supportive care alone as first‐line maintenance treatment for patients with locally advanced or metastatic urothelial carcinoma in Taiwan

**DOI:** 10.1002/cnr2.1887

**Published:** 2023-08-28

**Authors:** Po‐Jung Su, Ying Xiao, Amy Y. Lin, Connie Goh, Ethan Wu, Kevin Liu, Patrick Chou, Kaitlin Kuo, Roberto Palencia, Jane Chang, Mairead Kearney, Venediktos Kapetanakis, Agnes Benedict

**Affiliations:** ^1^ Department of Hematology‐Oncology, Chang Gung Memorial Hospital at Linkou and College of Medicine Chang Gung University Taoyuan Taiwan; ^2^ Evidera London UK; ^3^ Merck Ltd., Taipei, Taiwan, an affiliate of Merck KGaA Darmstadt Germany; ^4^ Pfizer Taiwan Ltd. Taipei Taiwan; ^5^ IQVIA Solutions Taiwan Ltd. Taipei Taiwan; ^6^ The Healthcare Business of Merck KGaA Darmstadt Germany; ^7^ Pfizer New York New York USA; ^8^ Evidera Budapest Hungary

**Keywords:** avelumab, economic model, health technology assessment, JAVELIN Bladder 100 study, Taiwan, urothelial carcinoma

## Abstract

**Background:**

Patients with locally advanced or metastatic urothelial carcinoma have limited treatment options and a poor prognosis. The JAVELIN Bladder 100 trial showed that avelumab as first‐line maintenance plus best supportive care significantly prolonged overall survival and progression‐free survival versus best supportive care alone in patients with locally advanced or metastatic urothelial carcinoma that had not progressed with first‐line platinum‐containing chemotherapy.

**Aims:**

We assessed whether avelumab plus best supportive care is a cost‐effective treatment option versus best supportive care alone in this patient group in Taiwan.

**Methods and Results:**

A partitioned survival model was used to estimate the costs and effects of avelumab plus best supportive care versus best supportive care alone over a 20‐year time horizon from the perspective of Taiwan's National Health Insurance Administration. Patient‐level data from JAVELIN Bladder 100 on efficacy, safety, utility, and time on treatment were analyzed to provide parameters for the model. Log‐normal and Weibull distributions were used for overall survival and progression‐free survival, respectively. Costs of healthcare resources, drug acquisition, adverse events, and progression were identified through publicly available data sources and clinician interviews. The model estimated total costs, life years, and quality‐adjusted life years. In the modeled base case, avelumab plus best supportive care increased survival versus best supportive care alone by 0.79 life years (2.93 vs. 2.14) and 0.61 quality‐adjusted life years (2.15 vs. 1.54). The incremental cost‐effectiveness ratio for avelumab plus best supportive care versus best supportive care alone was NT$1 827 680. Most (78%) of the probabilistic sensitivity analyses fell below three times the gross domestic product per capita. Scenario analysis indicated that life year and quality‐adjusted life year gains were most sensitive to alternative survival extrapolations for both avelumab plus best supportive care and best supportive care alone.

**Conclusion:**

Avelumab first‐line maintenance therapy combined with best supportive care was determined as a cost‐effective treatment strategy for patients in Taiwan diagnosed with locally advanced or metastatic urothelial carcinoma that had not progressed with platinum‐containing chemotherapy.

## 1 INTRODUCTION

Based on the Taiwan Cancer Registry annual report, in 2018 bladder cancer was the 11th most common cancer in Taiwanese males and 16th most common in Taiwanese females.^1^ In total, 4.57% of patients with bladder cancer were initially diagnosed with stage IV disease.^1^ Urothelial carcinoma (UC) accounted for 94.7% of bladder cancer cases in males (*n* = 1486) and 92.7% in females (*n* = 622).^1^ The 5‐year overall survival (OS) rate for patients with metastatic UC (mUC) was only 18% in Taiwan.^2^ The local Urology Management Guideline recommends first‐line (1L) treatment with methotrexate, vinblastine, doxorubicin, and cisplatin or gemcitabine plus cisplatin for patients with locally advanced UC or mUC (la/mUC).^3^ For those who are ineligible for cisplatin, recommended options include carboplatin, atezolizumab, or pembrolizumab.^3^ The National Comprehensive Cancer Network and European Society for Medical Oncology guidelines recommend cisplatin‐containing combination chemotherapy followed by avelumab maintenance as 1L treatment in eligible patients.^4^, ^5^ Carboplatin plus gemcitabine followed by avelumab maintenance is recommended in those with creatinine clearance between 30 and 60 mL/min or poor performance status.^4^, ^5^

Current 1L treatments for mUC that are eligible for reimbursement in Taiwan are carboplatin, cisplatin, and gemcitabine,^6^ which do result in good initial responses in most patients; however, chemotherapy can result in cumulative toxicities and lower quality of life, and associated improvements in progression‐free survival (PFS) and OS are not significant.^7^ According to von der Maase et al., the median time to disease progression was 7.4 months after 1L treatment with gemcitabine plus cisplatin in patients with la/mUC.^8^ Real‐world data from Taiwan showed that the median OS with cisplatin‐based and carboplatin‐based chemotherapy was 13.6 months for all patients and 7.2 months for patients over 70 years old.^9^

Avelumab is an anti–PD‐L1 human monoclonal antibody that was studied as a maintenance treatment following platinum‐containing chemotherapy in the JAVELIN Bladder 100 (JB100) trial. JB100 (NCT02603432) is the only multinational, multicenter, phase III clinical trial in the last 20 years to demonstrate a significant increase in OS with 1L treatment in patients with la/mUC.^10^ For patients with la/mUC who do not have disease progression after 4–6 cycles of 1L platinum‐containing chemotherapy, avelumab 1L maintenance therapy plus best supportive care (BSC) is associated with prolonged OS, increasing the 1‐year survival rate by 12.9% versus BSC alone (data cutoff, October 21, 2019; avelumab plus BSC, 71.3%; BSC alone, 58.4%).^10^ In long‐term follow‐up results from the JB100 trial (≥2 years in all patients; data cutoff, June 4, 2021), avelumab plus BSC significantly prolonged median OS by 8.8 months versus BSC alone (avelumab plus BSC, 23.8 months; BSC alone, 15.0 months; HR, 0.76 [95% CI: 0.63–0.91]; *p* = 0.0036).^11^ Avelumab plus BSC also prolonged median PFS by 3.4 months versus BSC alone (avelumab plus BSC, 5.5 months; BSC alone, 2.1 months; HR, 0.54 [95% CI: 0.46–0.64]; *p* < 0.0001).^11^

JB100 trial results provided category 1 evidence to support the inclusion of avelumab 1L maintenance in National Comprehensive Cancer Network, European Society for Medical Oncology, and European Association of Urology guidelines for patients with la/mUC that has not progressed with 1L chemotherapy.^4^, ^5^, ^12^ Avelumab received approval from the Taiwan Food and Drug Administration for the treatment of these patients.^13^ In addition, 95% of physicians who attended the Taiwan Urological Association consensus meeting in March 2021 agreed with prescribing avelumab as 1L maintenance therapy in patients without disease progression following 1L platinum‐containing chemotherapy.^14^ In Taiwan, 1L pembrolizumab and atezolizumab can only be reimbursed for patients who cannot tolerate chemotherapy and have tumors cells with high PD‐L1 expression.^6^ Because of this policy, between April 2019 and March 2021, only 84 Taiwanese patients with mUC in Taiwan received 1L immune checkpoint inhibitor (ICI) treatment.^15^ Hsieh et al. reported that 145 Taiwanese patients with la/mUC received at least one dose of ICIs from April 2019 to March 2020; 33 of these patients received 1L ICI treatment.^16^

This analysis was conducted to assess whether avelumab plus BSC is a cost‐effective treatment option versus BSC alone in patients with la/mUC that had not progressed with 1L platinum‐containing chemotherapy in Taiwan. A global partitioned survival model was adapted for Taiwan, and the cost‐effectiveness of avelumab plus BSC versus BSC alone was estimated in terms of the incremental cost‐effectiveness ratio (ICER), life years (LYs), and quality‐adjusted LYs (QALYs) over a 20‐year time horizon. The results were used to inform the decision on whether to offer reimbursement for avelumab plus BSC as a cost‐effective treatment option versus BSC alone from the perspective of the National Health Insurance Administration (NHIA) of Taiwan.

## 2 METHODS

### 2.1 Model overview

#### 2.1.1 Type of model

A global de novo partitioned survival model, handled in Microsoft Excel, of patients with la/mUC without disease progression following 1L platinum‐containing chemotherapy was built for adaptation to Taiwan.^17^, ^18^, ^19^ This model presented the analyses from the Taiwan payer perspective. This manuscript was written based on the ISPOR Consolidated Health Economic Evaluation Reporting Standards.^20^, ^21^

#### 2.1.2 Health states

**FIGURE 1:**
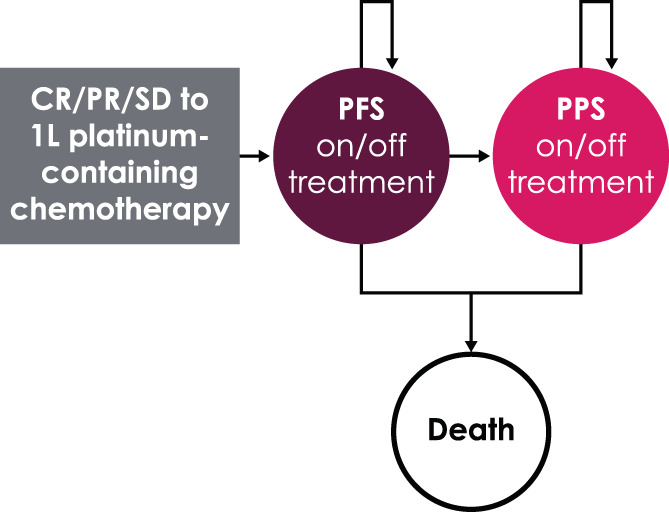
First‐line maintenance treatment in patients with locally advanced/metastatic urothelial carcinoma: Partitioned survival model structure. Arrows indicate transitions from one state to another. Arrows that curve back to the same state indicate that the patient remains in the same state. 1L, first line; CR, complete response; PFS, progression‐free survival; PPS, post‐progression survival; PR, partial response; SD, stable disease.

The conceptual structure considered three key mutually exclusive health states related to survival: PFS, post‐progression survival (PPS), and death (Figure 1). In this model, being on or off treatment in either the PFS or PPS health state was related to 1L maintenance treatment. In addition, we assumed that being on or off 1L maintenance treatment and subsequent treatments would only impact costs, not efficacy outcomes. Efficacy outcomes related to subsequent treatments were assumed to have been implicitly captured in OS data from JB100.

#### 2.1.3 Time horizon and cycle length

The model was adapted using a lifetime horizon of 20 years and a cycle length of 1 week to ensure that all important costs and outcomes were captured. This time horizon for estimating clinical and cost effectiveness sufficiently reflects differences in costs and outcomes between avelumab plus BSC and BSC alone. This time horizon was chosen following face‐to‐face interviews with Taiwanese urology and medical oncology experts during November 2020^22^ and was accepted by the Taiwanese Health Technology Assessment agency.^23^

#### 2.1.4 Type of analysis and discounting

Cost‐effectiveness and cost‐utility analyses were performed, and the results are presented as ICERs, which were expressed both as incremental cost per LY gained and per QALY gained. An annual discount rate of 3% was applied to both costs and QALYs, as stipulated in the Taiwanese Cost‐Effectiveness Analysis Guidelines.^23^

### 2.2 Patient populations

The model population comprised patients with la/mUC without disease progression following 1L chemotherapy. Patients enrolled in Asia made up 21% of the avelumab plus BSC arm in the overall JB100 population (75/350) and 23% of the BSC alone arm (81/350).^10^ The life table was updated with the Taiwanese version published by Taiwan's Ministry of the Interior.^24^ Table 1 presents patient characteristics for the study population.

**TABLE 1:** Patient characteristics.

Characteristics	Levels	Patient characteristics	Source
*N*		700	JAVELIN Bladder 100 (data on file)^10^
Age, years	Mean (standard deviation)	67.24 (9.51)	
First‐line regimen, %	Gemcitabine plus cisplatin	59.12	
	Gemcitabine plus carboplatin	40.88	
Sex, %	Male	77.29	
	Female	22.71	
Race, %	White	67.14	
	Asian	22.29	
	Black/African American	0.29	
	Other	5.14	
	Unknown	5.14	
Body weight, kg	Mean (standard deviation)	63.77 (16.23)	Taiwan National Nutrition Health Survey^28^
Body surface area, m^2^	Mean (standard deviation)	1.66 (0.24)	

### 2.3 Comparator treatment

The comparator treatment used in this model was BSC, which was also the comparator in the JB100 trial.^10^ The model was adapted during 2020, when no 1L maintenance treatments had been licensed for use or reimbursed for patients with la/mUC without disease progression after 1L chemotherapy in Taiwan. Subsequently, avelumab received regulatory approval for an indication extension to 1L maintenance treatment of la/mUC in Taiwan on April 1, 2021.

### 2.4 Clinical model parameters

#### 2.4.1 Efficacy

**FIGURE 2:**
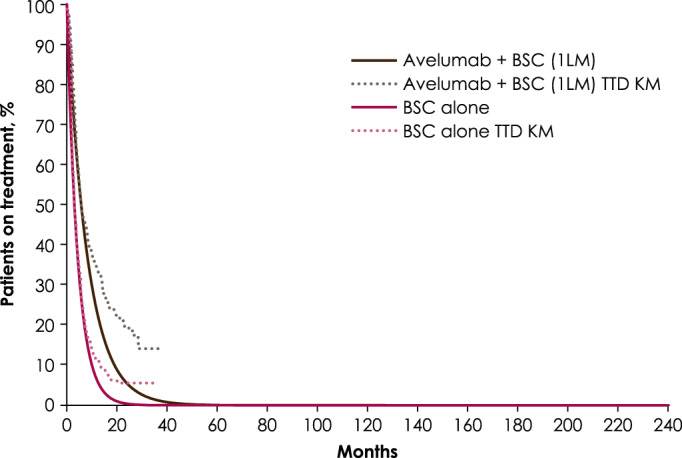
Projected time to treatment discontinuation for avelumab plus best supportive care versus best supportive care alone. The exponential distribution was chosen for avelumab. 1LM, first‐line maintenance; BSC, best supportive care; KM, Kaplan–Meier; TTD, time to treatment discontinuation.

Efficacy and safety data were obtained from the JB100 trial.^10^ The model extrapolated PFS and OS Kaplan–Meier curves from JB100 data^10^ to calculate the proportion of patients in each health state at a given time point and did not require explicit transition probabilities between health states. Time on treatment was modeled independently from PFS. We allowed patients to remain on treatment despite disease progression, consistent with JB100. The model has three options to model time on treatment, namely distribution best fitted to trial data, exponential distribution fitted to median treatment duration, and treatment until progression. As shown in Figure 2, the model is based on trial‐reported median treatment duration with exponential fit, which was validated by urology and medical oncology experts.^22^ Subsequent treatment effects were assumed to be captured in the OS data for avelumab plus BSC and BSC alone. The assumptions made for efficacy were validated in face‐to‐face interviews conducted in November 2020 with Taiwanese urology and medical oncology experts.^22^

#### 2.4.2 Utilities

Health utility data were collected in the JB100 trial using the EQ‐5D‐5L questionnaire. The EQ‐5D‐5L questionnaire is a standardized measure of health‐related quality of life that assesses a person's health status across five dimensions: mobility, self‐care, usual activities, pain/discomfort, and anxiety/depression. Each dimension has five levels of severity ranging from “no problems” to “extreme problems.” The utility values used for each health state in the model were estimated from a regression model, and data were collected using three covariates: baseline utility, progression status, and proximity to death.^25^ Coefficients for covariates are presented in Table 2. The fitted model has the form of an equation expressing mean utility as a sum of factors including an intercept, representing the baseline (patients with average utility at baseline who are progression free and had >30 days until death), and three other factors related to the effect of each covariate (progression, proximity to death, and baseline utility).

**TABLE 2:** Covariates for utility model including progression and proximity to death.

Covariates	Coefficients
EQ‐5D‐5L utility score at baseline (centered)	0.687
Progressed	−0.068
≤30 days until death	−0.202
Progression free and >30 days until death	0.776

The regression equation has the following form:

$$\text{Mean}\;\mathrm{EQ}‐\text{5D utility}=0.776+(-0.068\times \text{𝑥)}+(-0.202\times \text{𝑦)}+\left(0.687\times z\right)$$

where 𝑥 is “progressed,” 𝑦 is “≤30 days until death,” and *z* is “utility at baseline − average utility at baseline”

- “progressed” takes value 1 when a patient progresses and 0 when progression free
- “≤30 days until death” takes value 1 when a patient dies within the following 30 days and 0 when the patient does not die within the following 30 days
- “utility at baseline − average utility at baseline” takes value 0 for a person with average utility at baseline (centered baseline utility)
- 0.776 is the mean utility when all other factors in the equation are 0 (i.e., 0.776 is the mean utility for patients who are progression free, do not die within 30 days, and have an average utility at baseline)

Utilities were obtained using a crosswalk algorithm by Van Hout et al.^26^ for mapping EQ‐5D‐5L responses to EQ‐5D‐3L responses, along with the value set for EQ‐5D‐3L–derived weights from Dolan et al.^25^ No relevant local utilities were available from the global model; therefore, the utilities from the global model were used in this analysis.

### 2.5 Cost model parameters

#### 2.5.1 Maintenance treatment costs

The costs of the treatments used in the model were based on the NHIA list prices.^27^ All costs are presented in New Taiwan dollars (NT$) and are based on their 2020 value. The cost of BSC was assumed to be zero in both the intervention and comparator arms. To save on NHIA medication expenses, avelumab was assumed to be reimbursed—not at a flat price, but using weight‐based pricing. The model was adapted to the Taiwanese local environment using an average body weight of 63.8 kg, which was based on findings from the National Nutrition Health Survey^28^ and is lower than the 75 kg from the JB100 study.^22^

#### 2.5.2 Health care resource use

Treatment monitoring and disease management costs were based on health care resource use while on or off treatment in either the PFS or PPS health state, as well as a one‐off cost applied upon disease progression (Table 3). Unit costs were taken from NHIA medical services online.^29^

**TABLE 3:** First‐line maintenance treatment in patients with locally advanced or metastatic urothelial carcinoma: Health care resource use costs per model cycle.

Health states	Health care resource use categories	Cost input, NT$^a^	
		Avelumab plus best supportive care	Best supportive care alone
Progression‐free survival on/off treatment	On first‐line maintenance treatment	1059	220
	Off first‐line maintenance treatment	1192	1192
Post‐progression survival on/off treatment	Disease progression (one‐off)^b^	1286	1286
	Progressive disease (in addition to on/off treatment)	9259	9259
	On subsequent treatment	708	708

*Note*: Health care resource use estimates were initially obtained via a literature review and were validated by Taiwanese urology and oncology experts.

^a^ All costs are presented in NT$ and are based on their 2020 value.

^b^ One‐time cost applied upon disease progression.

#### 2.5.3 Subsequent treatment costs

The proportion of patients receiving active subsequent treatment was separately defined for avelumab plus BSC and BSC alone and was based on observations from JB100.^10^ We assumed that 68.52% of patients who had disease progression with avelumab maintenance treatment plus BSC would receive subsequent active treatments, whereas 86.06% of patients who had disease progression with BSC alone would receive subsequent active treatments (Table 4).^10^ We assumed that subsequent treatments would only affect drug costs. The basket of active subsequent treatments was also modeled separately for each treatment option and included cisplatin, carboplatin, docetaxel, paclitaxel, and pemetrexed. Under NHIA medications and reimbursement rule 9.69, only one ICI can be used for each indication, and it is not possible to switch ICI agents or combine the ICI with any targeted therapy.^30^ Additionally, if ICI treatment is ineffective, patients cannot switch to targeted therapy. Under this rule, the subsequent treatment received after avelumab plus BSC could not include any other ICIs, so its value in the model was set to 0%. Based on NHIA medications and reimbursement rule 9.69–2, which came into effect in 2020, all patients who received subsequent treatment following avelumab plus BSC were not permitted to receive any additional ICI treatment.^3^, ^30^ Patients who received subsequent active treatments incurred additional drug acquisition and administration costs. The distribution of subsequent treatments, which reflected the treatment landscape in Taiwan, was carefully reviewed by medical experts.

**TABLE 4:** Summary of subsequent treatments in the JAVELIN Bladder 100 trial and associated costs.

Input	Avelumab plus best supportive care	Best supportive care alone	Reference
Patients receiving subsequent treatment, %	68.52^a^	86.06^a^	JAVELIN Bladder 100^10^
Time on subsequent immune checkpoint inhibitor, weeks	16.68	25.12	
Time on subsequent standard chemotherapy, weeks	15.99	16.60	
Total costs for subsequent immune checkpoint inhibitor, NT$^b^	0	1 088 639	Calculation (National Health Insurance Administration medications website^27^)
Total costs for subsequent standard chemotherapy, NT$^b^	130 226	84 918	

*Note*: A model cycle is 1 week.

^a^ Data are in patients who had disease progression in either the avelumab plus best supportive care or best supportive care alone arm. These data differ from those reported in the JAVELIN Bladder 100,^10^ which correspond to subsequent treatment use in the overall population (47.7% and 65.1%, respectively).

^b^ All input costs are in NT$, based on 2020 values, and are applied at the time of disease progression (i.e., one‐time costs).

#### 2.5.4 Adverse events

The model included grade ≥3 adverse events (AEs) that occurred in more than 2% of patients in any arm of the JB100 trial,^10^ as these AEs were most likely to have the largest impact on the cost. The analysis assumed that the risk of an AE occurring was continuous while the patient was receiving treatment, which meant that the costs and utility decrements due to AEs were calculated per model cycle and were assessed for each cohort in each model cycle (Table 3).

#### 2.5.5 Adverse event costs

Costs related to AEs were calculated using an aggregated cost‐per‐event approach, which was based on the reported cost of inpatient stays or outpatient visits/care in the NHIA annual report.^31^ Estimates for AE management in either the inpatient or outpatient setting were obtained from face‐to‐face interviews conducted with Taiwanese urology and oncology experts^22^ (Table 5).

**TABLE 5:** Adverse events costs using aggregated costs per event.

	Unit cost (per event), NT$	Avelumab plus best supportive care^33^	Best supportive care alone^33^
Duration of treatment exposure, weeks	–	24.90	13.10
Urinary tract infection	1099	6.00%	3.80%
Increased alkaline phosphatase	483	2.90%	2.30%
Anemia	927	4.40%	3.20%
Hyponatremia	558	6.00%	2.60%
Lipase increased	483	8.00%	6.00%
Amylase increased	483	5.00%	1.80%
Immune‐mediated pneumonitis	53 162	0.50%	0%
Immune‐mediated hepatitis	11 517	0.70%	0%
Immune‐mediated colitis	8677	0.40%	0%
Immune‐mediated adrenal insufficiency	558	0.10%	0%
Immune‐mediated hypothyroidism	558	0.19%	0%
Immune‐mediated hyperthyroidism	558	0.01%	0%
Immune‐mediated type 1 diabetes mellitus	558	0.10%	0%
Immune‐mediated nephritis	18 639	0.10%	0%

*Note*: The table shows grade ≥3 adverse events that occurred in ≥2% of patients and all grade ≥3 immune‐related adverse events that occurred in either treatment arm.

#### 2.5.6 Terminal care costs

Terminal care costs, including medication expenses, diagnostic and treatment costs, procedure costs, and radiation costs, were obtained from the NHIA Annual Monitoring Report, which summarized hospice costs in the previous 6 months.^32^

### 2.6 Clinical and economic validation

The assumptions used for efficacy were validated in face‐to‐face interviews with urology and oncology mUC experts from Taiwan that took place in November 2020.^22^ Health care resource use before and after disease progression, AE management, and patterns of subsequent therapy were validated during the interviews, and it was confirmed that the parameters and treatment pathway used in the model aligned with clinical practice in Taiwan.

### 2.7 Sensitivity analyses

#### 2.7.1 One‐way sensitivity analysis

One‐way sensitivity analysis was conducted to assess the sensitivity of cost‐utility results to individual parameters associated with uncertainty in the model (Table 6). The parameters were population age at treatment initiation, percentage of male patients, efficacy (according to OS and PFS data), median duration of treatment with avelumab plus BSC or BSC alone, percentage of patients receiving subsequent treatment after avelumab plus BSC or BSC alone, time on subsequent ICI or standard chemotherapies (cisplatin, carboplatin, docetaxel, paclitaxel, and pemetrexed), total AE costs, and health state utility.

**TABLE 6:** One‐way sensitivity analysis parameters for avelumab plus best supportive care versus best supportive care alone.

Parameter	Base case	Lower value	Upper value
Population age at treatment initiation, years	67.50	66.81	68.19
Proportion of male patients, %	77	74	81
Proportion of female patients, %	23	19	26
Efficacy—first‐line maintenance overall survival HR: best supportive care alone	1.33	0.99	1.79
Efficacy—first‐line maintenance progression‐free survival HR: best supportive care alone	1.47	1.20	1.79
Median treatment duration: avelumab plus best supportive care (first‐line maintenance), weeks	24.90	22.41	27.39
Median treatment duration: best supportive care alone (first‐line maintenance), weeks	13.10	11.79	14.41
Proportion of patients receiving subsequent treatment: avelumab plus best supportive care (first‐line maintenance), %	68.52	60.84	75.73
Proportion of patients receiving subsequent treatment: best supportive care alone (first‐line maintenance), %	86.06	81.14	90.34
Time on subsequent immune checkpoint inhibitor (first‐line maintenance population): avelumab plus best supportive care (first‐line maintenance), weeks	16.68	9.58	23.77
Time on subsequent immune checkpoint inhibitor (first‐line maintenance population): best supportive care alone (first‐line maintenance), weeks	25.12	20.57	29.67
Time on subsequent standard chemotherapies (first‐line maintenance population): avelumab plus best supportive care (first‐line maintenance), weeks	15.99	14.24	17.74
Time on subsequent standard chemotherapies (first‐line maintenance population): best supportive care alone (first‐line maintenance), weeks	16.60	14.66	18.54
Total adverse event cost: avelumab plus best supportive care (first‐line maintenance), per model cycle, NT$	25.15	22.64	27.67
Total adverse event cost: best supportive care alone (first‐line maintenance), per model cycle, NT$	10.47	9.42	11.52
Health state utility: progression free, >30 days until death	0.78	0.76	0.79
Health state utility: progression free, ≤30 days until death	0.57	0.53	0.62
Health state utility: post progression, >30 days until death	0.71	0.69	0.72
Health state utility: post progression, ≤30 days until death	0.51	0.46	0.55

#### 2.7.2 Probabilistic sensitivity analysis

A probabilistic sensitivity analysis was undertaken to explore the joint uncertainty of all model parameters and their associated impact on cost‐utility results, and it was performed by running 1000 iterations. A normal distribution was used for population age at treatment initiation as the sample size of this cohort is more than 30; a beta distribution was used for the percentage of male patients, percentage of patients receiving subsequent treatment, and health state utility; the log‐normal distribution was used for efficacy; and a gamma distribution was used for median treatment duration, time on subsequent ICI or standard chemotherapy, total AE costs, subsequent treatment cost, and health care resource use cost per model cycle.

## 3 RESULTS

### 3.1 Base‐case results

Avelumab plus BSC increased survival versus BSC alone by 0.79 LYs (2.93 vs. 2.14) and 0.61 QALYs (2.15 vs. 1.54). The total LYs and QALYs in the PFS, PPS, and death states with avelumab plus BSC were higher than those with BSC alone. Most costs were higher for avelumab plus BSC, except for costs such as subsequent treatment (NT$69 460 vs. NT$862 798) and terminal care costs (NT$63 421 vs. NT$65 368), which were higher for BSC alone (Table 7).

**TABLE 7:** Avelumab plus best supportive care versus best supportive care alone: Effectiveness and cost results.

	Avelumab plus best supportive care	Best supportive care alone
Total life years	2.93	2.14
Progression‐free survival	1.38	0.69
Post‐progression survival	1.55	1.45
Time on treatment	0.68	0.36
Total quality‐adjusted life years	2.15	1.54
Progression‐free survival	1.06	0.53
Post‐progression survival	1.09	1.01
Costs, NT$		
Drug acquisition cost (first‐line maintenance)	1 778 939	0
Drug administration cost (first‐line maintenance)	17 949	0
Adverse event management cost (first‐line maintenance)	840	180
Disease progression cost (one‐off)	1251	1650
Health care resource use	887 653	784 265
Subsequent treatment cost	69 460	862 798
Terminal care costs	63 421	65 368

**FIGURE 3:**
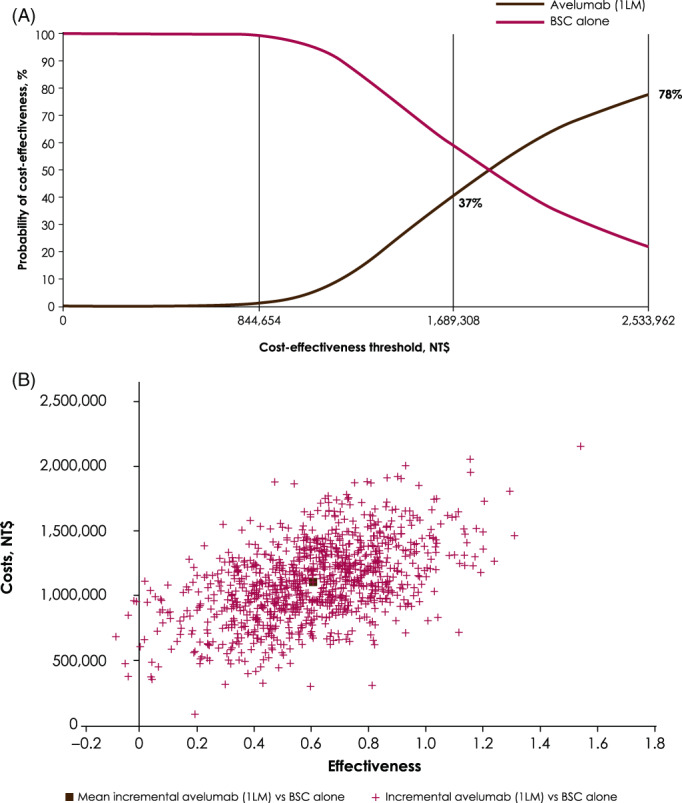
Cost‐effectiveness for avelumab plus best supportive care versus best supportive care alone: (A) acceptability curve; (B) plane. 3 × GDP = NT$2 533 962; 2 × GDP = NT$1 689 308; current Taiwan GDP = NT$844 654. 1LM, first‐line maintenance; BSC, best supportive care; GDP, gross domestic product.

A willingness to pay threshold of three times the gross domestic product (GDP) per capita was adopted, according to the World Health Organization definition.^34^ The ICER for avelumab plus BSC versus BSC alone was NT$1 827 680, which was less than three times the GDP per capita^35^ (Figure 3A).

### 3.2 Probabilistic sensitivity analysis

The probabilistic sensitivity analysis was performed using 1000 repetitions, and the results are presented in a scatter plot (Figure 3B). The results showed the uncertainty in the estimates of expected incremental cost and expected incremental effect (QALYs gained) when comparing avelumab plus BSC with BSC alone (Figure 3B). Overall, 37% and 78% of the probabilistic sensitivity analyses fell within two and three times the GDP per capita cost‐effectiveness thresholds, respectively (Figure 3A).

### 3.3 One‐way sensitivity analysis

**FIGURE 4:**
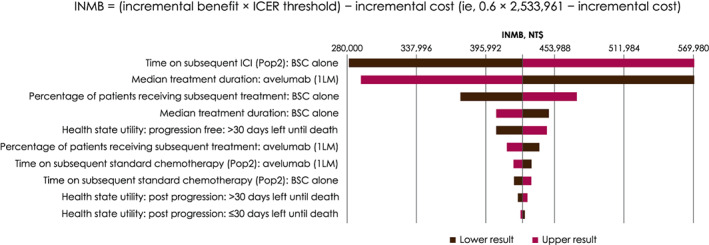
Tornado diagram of the incremental net monetary benefit of avelumab plus best supportive care versus best supportive care alone. The vertical line in the center indicates the incremental net monetary benefit of the base‐case scenario. The darker bar indicates the incremental net monetary benefit result when the minimum input value is used, while the pink bar indicates the incremental net monetary benefit result when the maximum input value is used. 1LM, first‐line maintenance; BSC, best supportive care; ICER, incremental cost‐effectiveness ratio; ICI, immune checkpoint inhibitor; INMB, incremental net monetary benefit; Pop2, first‐line maintenance population.

A one‐way sensitivity analysis showed that the incremental net monetary benefit (INMB) decreased when values of the following parameters increased: time on subsequent ICI following progression with BSC alone and the percentage of patients receiving subsequent treatment following BSC alone. However, the INMB increased when the median treatment duration of avelumab plus BSC increased. In this model, INMB equaled incremental benefit times ICER threshold, which is 0.6 × 2 533 961 − incremental cost. Varying health state utilities by ≤0.02 did not have a significant impact on the INMB (Table 5; Figure 4).

### 3.4 Scenario analysis

Since the NHIA has a limited budget for the reimbursement of new medications, confidential agreements can be contracted with pharmaceutical companies. In a hypothetical scenario, a rebate of 20% of the negotiated price of avelumab would yield an even lower ICER of NT$1 239 338.

## 4 DISCUSSION

The results of this analysis, which was adapted from the global cost‐effectiveness model, are consistent with other published results. For example, the ICER in our analysis is less than three times the GDP per capita, which is similar to findings in countries such as Finland.^18^ In the Finnish study, cost‐effectiveness analysis for avelumab plus BSC versus BSC alone as 1L maintenance treatment in patients with la/mUC showed that the ICER was less than three times the GDP per capita.^18^ In a study conducted in the United Kingdom, avelumab plus BSC 1L maintenance treatment showed potential cost effectiveness because avelumab plus BSC was associated with longer survival than BSC alone.^19^

Based on the findings of Peng et al., avelumab plus BSC may be a cost‐effective option for patients with la/mUC in the United States at a willingness to pay threshold of US$150 000 per QALY.^36^ However, in Lin et al. study, avelumab maintenance therapy is considered cost ineffective with the same population at a willingness to pay threshold of US$200 000 per QALY.^37^ These conflicting results may, in part, be due to different assumptions in utility and subsequent treatments used in the respective analyses. The utility information of Peng et al.^36^ and Lin et al.^37^ are taken from different literatures sources and assumed avelumab's utility values are similar to those of pembrolizumab^38^ and nivolumab.^39^ To ensure greater accuracy, this study uses the utility data of avelumab plus BSC collected in the JB100 clinical trial.

The subsequent treatment assumptions used in Peng et al.^36^ and Lin et al.^37^ are taken from JB100. In contrast, and to reflect the reimbursement policy in Taiwan,^30^ in our study all patients who received subsequent anticancer treatment following avelumab plus BSC were assumed to have received chemotherapy.^30^ The choice and cost of subsequent treatment were important drivers in all the cost‐effectiveness models because a higher percentage of patients who had disease progression with BSC alone received more costly subsequent ICI treatment than those who had disease progression with avelumab plus BSC. Indeed, long‐term data in all patients with ≥2‐years of follow‐up from JB100 demonstrated that the majority of patients received chemotherapy as a subsequent drug therapy following avelumab plus BSC.^11^

Subsequent treatment following disease progression with avelumab plus BSC or BSC alone and time to treatment discontinuation were the top two drivers in this cost‐effectiveness analysis model. Time to treatment discontinuation strongly influenced cost‐effectiveness values and results for avelumab. The model, adapted to Taiwan, used the exponential distribution based on the median time to treatment discontinuation in both the avelumab plus BSC and BSC alone arms. Scenario analyses showed that one of the most important drivers of cost effectiveness was the choice of statistical distribution used to extrapolate PFS, and this could also influence the model results. The Weibull distribution provided a more conservative assumption because it predicted a lower PFS at the tail of the Kaplan–Meier curve than the log‐normal distribution; therefore, this distribution was selected as the reference, and was confirmed by Taiwanese clinical experts. Compared with the Weibull distribution, choosing a log‐normal distribution to extrapolate PFS caused an approximate 0.2% increase in the total cost and an approximate 0.9% increase in the total QALYs in the avelumab plus BSC arm.

This economic model adaptation has several strengths. First, the analyses and predictions were based on the results of the JB100 trial, which was a large, international, randomized controlled trial with an appropriate comparator that was generalizable to Taiwanese clinical practice. Second, patients of Asian race in the JB100 trial accounted for 21% of the avelumab plus BSC arm (75/350) and 23% of the BSC alone arm (81/350),^10^ which means JB100 included a sizeable Asian subgroup. Third, validation by clinical experts was performed to mitigate areas of uncertainty within the cost‐effectiveness analysis and to select representative base‐case assumptions for Taiwan. Lastly, extensive scenario analyses were conducted using different survival curve extrapolation options to interpret clinical uncertainty in the modeled survival.

Like other economic analyses, this cost‐effectiveness analysis was subject to limitations. We used several assumptions to build the economic model to simulate the costs and effectiveness of avelumab 1L maintenance therapy plus BSC. The impact of these assumptions and inherent uncertainty was tested in several sensitivity and scenario analyses that showed the robustness of our findings. Notwithstanding, the efficacy data in this analysis were based on OS and PFS data from JB100,^10^ and, at the time of this analysis, other real‐world effectiveness outcomes were yet to be published. Moreover, the model relied on projections of PFS and OS beyond the JB100 study period. PFS and OS projections for BSC alone were verified with external data, but long‐term outcomes for avelumab plus BSC could not be verified. Additionally, only the costs of subsequent treatments were considered, neglecting any possible further impact of these treatments on clinical and safety outcomes. AEs associated with subsequent treatments were not modeled because they were not expected to have a significant impact on the results. Based on interviews conducted in November 2020, clinical experts described patients who respond to 1L chemotherapy in Taiwan as having a higher frequency of cisplatin use and better renal function. Lastly, no relevant local utilities were available for use in the model, so utilities based on the JB100 intention‐to‐treat population were used in this analysis.

The analysis demonstrated the likely cost effectiveness of avelumab 1L maintenance as a standard of care in this setting both in cisplatin‐eligible and cisplatin‐ineligible patients and irrespective of PD‐L1 status in Taiwan. The use of avelumab 1L maintenance may be incorporated into current treatment guidelines for la/mUC.^40^, ^41^ Patients who are eligible for 1L platinum‐containing chemotherapy may be considered for avelumab 1L maintenance if they have not had disease progression following  chemotherapy. Additionally, healthcare providers and policy makers may consider the cost‐effectiveness of avelumab when making decisions about treatment options and healthcare resource allocations.

## 5 CONCLUSION

It is observed that avelumab plus best supportive care costs NT$2 819 513 and best supportive care alone costs NT$1 714 261. Considering the significantly improved outcomes with avelumab plus best supportive care, this treatment option can be considered more cost effective than best supportive care alone. Avelumab first‐line maintenance therapy with best supportive care may be considered a cost‐effective treatment strategy for patients with locally advanced or metastatic urothelial carcinoma that has not progressed on or after first‐line platinum‐containing chemotherapy in Taiwan based on National Health Insurance Administration medications and reimbursement rule.

## AUTHOR CONTRIBUTIONS

**Po‐Jung Su:** Writing – original draft (equal); writing – review and editing (equal). **Ying Xiao:** Conceptualization (equal); data curation (equal); formal analysis (equal); methodology (equal); writing – original draft (equal); writing – review and editing (equal). **Amy Y. Lin:** Writing – original draft (equal); writing – review and editing (equal). **Connie Goh:** Writing – original draft (equal); writing – review and editing (equal). **Ethan Wu:** Conceptualization (equal); methodology (equal); writing – original draft (equal); writing – review and editing (equal). **Kevin Liu:** Conceptualization (equal); methodology (equal); writing – original draft (equal); writing – review and editing (equal). **Patrick Chou:** Conceptualization (equal); data curation (equal); formal analysis (equal); methodology (equal); supervision (equal); writing – original draft (equal); writing – review and editing (equal). **Kaitlin Kuo:** Conceptualization (equal); data curation (equal); formal analysis (equal); methodology (equal); resources (equal); writing – original draft (equal); writing – review and editing (equal). **Roberto Palencia:** Conceptualization (equal); formal analysis (equal); project administration (equal); supervision (equal); writing – original draft (equal); writing – review and editing (equal). **Jane Chang:** Conceptualization (equal); formal analysis (equal); methodology (equal); supervision (equal); writing – original draft (equal); writing – review and editing (equal). **Mairead Kearney:** Conceptualization (equal); formal analysis (equal); methodology (equal); supervision (equal); writing – original draft (equal); writing – review and editing (equal). **Venediktos Kapetanakis:** Conceptualization (equal); data curation (equal); formal analysis (equal); methodology (equal); writing – original draft (equal); writing – review and editing (equal). **Agnes Benedict:** Conceptualization (equal); data curation (equal); formal analysis (equal); methodology (equal); writing – original draft (equal); writing – review and editing (equal).

## CONFLICT OF INTEREST STATEMENT

Po‐Jung Su has nothing to disclose. Venediktos Kapetanakis is an employee of Evidera, London, UK. Ying Xiao was an employee of Evidera, London, UK at the time the analysis was conducted. Amy Y. Lin was an employee of Merck Ltd., Taipei, Taiwan, an affiliate of Merck KGaA, Darmstadt, Germany, at the time the analysis was conducted. Connie Goh is an employee of Merck Ltd., Taipei, Taiwan, an affiliate of Merck KGaA, Darmstadt, Germany. Ethan Wu and Kevin Liu are employees of Pfizer, Taipei, Taiwan. Patrick Chou and Kaitlin Kuo are employees of IQVIA Solutions Taiwan Ltd., Taipei, Taiwan. Roberto Palencia was an employee of the healthcare business of Merck KGaA, Darmstadt, Germany at the time the analysis was conducted. Mairead Kearney is an employee of the healthcare business of Merck KGaA, Darmstadt, Germany. Jane Chang is an employee of Pfizer, New York, NY, USA. Agnes Benedict is an employee of Evidera, Budapest, Hungary.

## ETHICS STATEMENT

Institutional ethics approval and patient consent were not required for this study.

## Data Availability

The datasets generated and analyzed during the current study are available from the corresponding author upon reasonable request.
